# The True Impact of Voiding Dysfunctions after Transobturator Sub-Urethral Tape Procedures: A Systematic Review of Literature

**DOI:** 10.3390/jcm13164762

**Published:** 2024-08-13

**Authors:** Francesco Plotti, Stefania Rampello, Corrado Terranova, Carlo De Cicco Nardone, Daniela Luvero, Roberto Montera, Violante Di Donato, Anna Franca Cavaliere, Giuseppe Campagna, Fernando Ficarola, Arianna Martinelli, Roberto Angioli

**Affiliations:** 1 Research Unit of Gynaecology, Department of Medicine and Surgery, Università Campus Bio-Medico di Roma, 00128 Rome, Italy; 2 Fondazione Policlinico Universitario Campus Bio-Medico, Via Alvaro del Portillo 200, 00128 Rome, Italy; 3 Division of Obstetrics and Gynecology, Ospedale Isola Tiberina Gemelli Isola, 00186 Rome, Italy; 4 Department of Gynecological, Obstetrical and Urological Sciences, “Sapienza” University of Rome, 00185 Rome, Italy; 5 Obstetrics and Gynecological Unit, Department of Woman’s and Child’s Health, San Camillo-Forlanini Hospital, 00152 Rome, Italy

**Keywords:** transobturator tape, TOT, TVT-O, voiding dysfunctions, de novo OAB, urinary tract infections

## Abstract

**Introduction**: Transobturator techniques are frequently used for the surgical treatment of female stress urinary incontinence (SUI), due to their high success rates and few intraoperative complications. However, controversial results have been reported in the literature regarding their incidence. The aim of this study is to analyze the real incidence and trend over time of such complications, especially voiding dysfunctions and overactive bladder (OAB) symptoms. **Methods**: A comprehensive search using PubMed/MEDLINE, Scopus, and Cochrane databases was performed. The search string used was the following: (female stress urinary incontinence) AND (complication) AND ((midurethral sling) OR (transobturator tape) OR (TVT-O) OR (voiding dysfunctions) OR (de novo OAB) OR (recurrent UTI) OR (vaginal erosion)). We included randomized controlled trials, prospective controlled studies, prospective and retrospective observational studies. All selected articles were screened based on titles and abstracts. Relevant data were extracted and tabulated. **Results**: A total of 39 studies were included in our analysis. Transobturator tape procedures show a high objective cure rate for SUI, from 76.9% to 100%. Postoperative voiding dysfunctions are shown to be quite common, ranging from 0–22% of cases. Despite that, this percentage decreases to 0–1% after 12 months. De novo OAB incidence ranges from 3% to 14% at 12 months, with variability over time due to multiple factors. Tape-related complications usually occur after 12 months, with a variable incidence up to 7%. Urinary tract infections (UTIs) are quite common in the immediate postoperative period but sometimes can be recurrent, requiring long-term prophylactic antibiotic treatment. **Conclusions**: Voiding dysfunctions are generally transient complications, while de novo OAB may persist over time. An adequate preoperative counseling, along with accurate written informed consent, could enhance patient tolerance of these issues and contribute to long-term patient satisfaction.

## 1. Introduction

Urinary incontinence (UI) is defined as a complaint of involuntary loss of urine [1]. UI affects women globally, impacting not only their physical well-being but also their psychological and socio-economic status [2]. Three types of UI can be defined: stress urinary incontinence (SUI), urge urinary incontinence (UUI) and mixed urinary incontinence (MUI). According to Hunskaar et al., SUI is the most common, with a prevalence of 49% in non-institutionalized women [3]. First-line treatment in SUI is usually conservative. In case of conservative treatment failure, a surgical approach is often considered. Among the surgical techniques, retropubic and transobturator mid-urethral slings are most frequently used, because of their higher success rates and shorter operative time. A Cochrane review showed that over 80% of women with stress urinary incontinence have been cured or had a significant improvement in their symptoms, irrespective of the sling used and the route of sling insertion [4]. Transobturator techniques are nowadays the most commonly used because of their safety, compared to other techniques [5,6,7]. Transobturator techniques include the outside-in transobturator tape (TOT) technique, the inside-out tension-free vaginal tape-obturator (TVT-O) technique, and its modified version, the TVT-Abbrevo technique.

Despite having many advantages, certain complications can arise, such as voiding dysfunctions and de novo overactive bladder (OAB). These complications lead to a decrease in patients’ quality of life (QoL) and subjective success rate [8]. The International Continence Society (ICS) and the International Urogynecological Association (IUGA) define a ‘Voiding dysfunction’ (VD) by symptoms and urodynamic investigations. In particular, urodynamic investigation shows an abnormally slow and/or incomplete micturition, resulting in high post-void residuals. Patients with voiding dysfunctions may exhibit various symptoms. Voiding symptoms include hesitancy, slow stream, intermittency, straining to void, splitting of the urinary stream, position-dependent micturition and the feeling of incomplete bladder emptying [9]. Various treatment options can be adopted, based on symptom duration: short-term self-catheterization (0–48 h symptoms duration), long-term self-catheterization and tape mobilization or loosening in more persistent cases [10]. Currently, there is no consensus in the literature regarding postoperative voiding dysfunction incidence after performing transobturator techniques. The aim of this systematic review is to analyze the real incidence and trend over time of postoperative voiding dysfunctions after transobturator techniques. Secondarily, it investigates the incidence of its related complications, such as de novo urgency/OAB, vaginal erosions, tape extrusions and low urinary tract infections.

## 2. Materials and Methods

The study was conducted in accordance with the requirements of the Preferred Reporting Items for Systematic Reviews and Meta-Analyses (PRISMA 2020) review protocol. The PRISMA statement comprises a 27-item checklist covering review contents and a four-phase flow diagram documenting the study selection process [11]. The study was approved by our institution Internal Review Board. A literature search was performed independently by two researchers (S.R. and F.P.) using Pub-Med/MEDLINE, Scopus and the Cochrane Library. All articles in the English language have been included without restrictions on the date of publication. The PICOS (Population, Intervention, Comparison, Outcomes and Study) framework was used to assess the eligibility criteria of our articles. We used the following search string: (female stress urinary incontinence) AND (complication) AND ((midurethral sling) OR (transobturator tape) OR (TVT-O) OR (voiding dysfunctions) OR (de novo OAB) OR (recurrent UTI) OR (vaginal erosion)). We also reviewed the reference list in the selected articles to search for further eligible studies fulfilling the inclusion criteria. Studies included in our analysis met all the following criteria: randomized controlled trials or prospective controlled studies or prospective/retrospective observational studies; studies on human subjects; studies reporting voiding dysfunctions and other complications related to transobturator tape procedures. Systematic reviews, review articles, case reports, case series and letters to the editor were excluded from the review. Studies including patients with preoperative mixed urinary incontinence—if specified—were also excluded. Two investigators (S.R and F.P.) independently selected the studies to be included in the results by reading titles and abstracts. Any discrepancies in the selection process were resolved through discussion to reach a consensus. The same investigators read the full-text manuscripts and extracted the following information from each study: authors’ names, year of publication, type of study, number of participants, aim of the study, surgical details of the procedure, general information about recruited patients (type of incontinence, preoperative urgency), follow-up time, objective cure rate, and postoperative complications. The cure rate was determined heterogeneously among the studies: some studies determined it by urodynamic evaluation, other studies by stress test or pad test. The postoperative complications reported in this study were: voiding symptoms, tape-related complications (such as vaginal erosion or tape exposure/extrusion), de novo OAB, detrusor overactivity (DO), urgency incidence after surgery and postoperative urinary tract infection. With regard to the risk-of-bias assessment, no specific tool was used, as the expected heterogeneity in study design would not allow the use of a single tool for all datasets and thus a comparison of the risk of bias between studies. However, there was a group discussion among the authors about potential biases in the reviewed studies, and identified sources of bias are presented in our results and discussion.

## 3. Results

A literature search based on the above-mentioned string initially yielded 2301 articles. We excluded from the list systematic reviews, review articles, case reports, case series, and letters to the editor, leaving 1114 records remaining. Additionally, 18 more papers were obtained from a cross-reference search of some of these articles.

**Figure 1:**
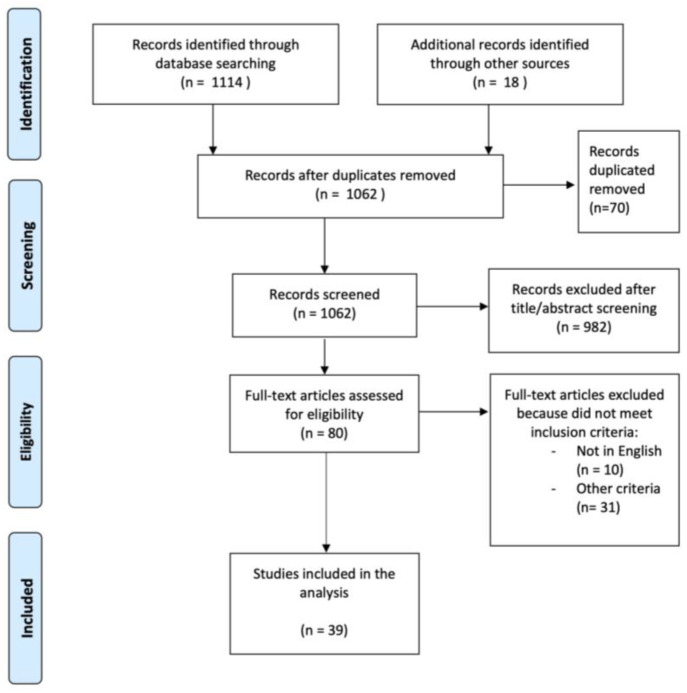
PRISMA flow-chart.

After removing 70 duplicates, 982 articles were excluded due to title or abstract screening. Subsequently, 41 full-text articles were excluded for not meeting the inclusion criteria. Finally, 39 studies that met the eligibility criteria were included in the present review. These studies comprised of 22 randomized controlled trials, 7 prospective studies, 9 retrospective studies and 1 bidirectional study (both prospective and retrospective). The following PRISMA flow chart (Figure 1) visually represents the search and study selection process.

The results are summarized in Table S1. In our analysis, voiding dysfunctions were reported in 22 studies, tape-related complications in 16 studies, de novo urgency or overactive bladder (OAB) in 16 studies, and urinary tract infections (UTIs) in 11 studies.

Transobturator tape procedures show a high objective cure rate for SUI, between 76.9% and 100%. Voiding dysfunction or symptoms are quite common after these procedures, affecting a variable percentage of patients, ranging from 0–22%. Transient urinary retention is common in the immediate postoperative period and is often treated with temporary catheterization. Sometimes, in the case of symptoms persistence, prolonged catheterization or tape release are the preferred treatment options.

Only eight studies analyze voiding dysfunction incidence in the long-term follow-up (more than 12 months). In particular, they report a decreasing trend of voiding dysfunction over time, in some cases as low as 0–1% [8,12,13,14,15,16,17,18]

Another frequent complication is the development of postoperative de novo OAB, which occurs in about 3–14% of cases at 12 months.

Lower urinary tract infections (UTIs) occur in most cases immediately after surgery and usually resolve with antibiotic treatment. Few cases of recurrent UTIs after 12 months are reported, often treated with long-term prophylactic antibiotics [14,15,19].

Tape-related complications, such as vaginal erosion with or without tape exposure, occur after 12 months in most studies with a variable incidence of up to 7%.

## 4. Discussion

The literature research performed shows us that almost all authors agree on the objective cure rate of transobturator techniques, typically exceeding 90%. This result is comparable to the retropubic procedure. Serati et al., in a 13-year follow-up study, found that 90.9% of patients were objectively cured, with urodynamic confirmation in 89.1% of cases [20].

On the other hand, there is not a clear consensus on lots of aspects regarding transobturator technique postoperative complications.

For convenience, we analyze the results of the complications researched by dividing them into separate paragraphs.

***a.***  Voiding disfunction

Both retropubic and transobturator techniques are equally effective. The first is associated with more intraoperative complications, such as bladder perforation. Thus, cystoscopy is mandatory. Moreover, transobturator techniques are not complication-free, and among those, voiding dysfunctions are one of the most frequent. VD symptoms can vary in type and severity, ranging from a feeling of incomplete emptying to prolonged urinary retention [9]. Lemack et al. affirmed that bladder outlet obstruction is often iatrogenic, following traditional anti-incontinence procedures, with an incidence of urethral obstruction of up to 20%. Regarding specific procedures, Lemack et al. also noted that tension-free vaginal tape (TVT) is associated with a lower incidence of voiding dysfunctions, compared to other traditional anti-incontinence procedures. However, there were insufficient data available regarding transobturator tape procedures [21].

Morey et al. found that the incidence of transient voiding dysfunction is similar in both retropubic and transobturator approaches, while the long-term risk of voiding dysfunction is lower in patients who undergo transobturator tape treatment [22,23].

The most heterogeneous data found were those concerning the postoperative complications rate among different transobturator techniques.

Liapis et al. reported, at 12 months, voiding disfunction incidence in 5% of patients who underwent TVT-O and 3.8% in those who underwent TOT. Scheiner et al. found a 13.5% incidence of voiding dysfunctions after TVT-O at 12 months, compared to 11.8% after TOT. Wen-Chen Huang et al. reported a similar incidence of 11.2% at 12 months after TOT [17,24,25].

These results could be influenced by the poor homogeneity among studies, especially regarding follow-up time and patient selection. For example, many studies included patients with MUI, OAB or DO. Consequently, postoperative results and patient global satisfaction could be influenced by the presence of these additional problems.

The etiology of postoperative voiding dysfunctions is probably related to the hypersuspension or kinking of the urethra. This condition could be provoked by naïve surgeons tendency to hypercorrection, or by periurethral scarring after inadvertent suture placement through the urethral lumen, or by excessive surgical dissection [26]. Since the success of this surgery depends almost exclusively on the tension used, if the tape is correctly positioned the chance of success is higher [8].

For this reason, the surgeon’s learning curve is crucial to establish the correct tape tension. Montera et al. described that at least ten procedures were necessary for a ‘naïve’ surgeon to learn the TVT-O technique, in order to reduce operative times while ensuring both safety and good outcomes for patients with SUI [27].

Transient urine retention can also occur due to edema, inflammation and liquid congestion in urethral and bladder neck tissues following an anti-incontinence procedure. This alteration disrupts the equilibrium during the bladder emptying phase [28]. This condition is called ‘bladder–sphincter dyssynergia’ and results from the new resistance system introduced by the transobturator sling placement. Before surgery, the bladder outlet resistance is lower, but the presence of the sling increases it, potentially leading to bladder overdistension and detrusor injury. Surgeon overcorrection, iatrogenic fibrosis, and urethral stricture can further exacerbate this obstruction, resulting in a detrusor hypotonia and slower contraction velocity compared to the norm [29].

This study indicates that voiding dysfunctions are often transient, likely due to inflammation and tissue edema or temporary conditions resulting from the surgical procedure.

As concerns bladder–sphincter dyssynergia, after the initial detrusor hypotonia due to bladder outlet obstruction (BOO), detrusor contraction power gradually increases to maintain voiding. Oalke et al. observed continuous increases in detrusor contraction power parameters with rising BOO grade in adult male patients with lower urinary tract symptoms (LUTS) [30]. Some ultrasound studies have also demonstrated bladder wall hypertrophy in adult men with BOO. The thickening of the bladder wall (contractile elements) contributes to increased detrusor contraction power [31,32].

Additionally, Holm et al. correlated ultrastructural findings with urodynamic parameters in BOO patients, revealing that myohypertrophy patterns are not constant. However, a significant association between elastosis and the degree of obstruction was evident. Elastosis secondary to BOO may enhance the compliance of the obstructed detrusor [33].

However, detrusor response to BOO is also influenced by age. Sheng-Fu Chen et al. affirmed that there is an age-related impairment of contractility associated with structural changes in the detrusor muscle (decreased axonal content, increased collagen content, and changes in muscarinic receptors). Specifically, the increasing detrusor collagen content (fibrosis) is associated with decreased bladder compliance, DO, and urinary retention [34].

Therefore, we can affirm that the restoration of bladder–sphincter equilibrium after transobturator tape procedures is a multifactorial process. Thus, voiding dysfunctions due to bladder–sphincter dyssynergia sometimes regress, but other times persist throughout life, especially in older patients. Finally, when the etiology of postoperative voiding dysfunctions is iatrogenic (due to hypercorrection by naive surgeons), the only treatment option is surgery with tape mobilization or tape loosening to achieve a more desirable tension without interfering with urinary flow [22].

***b.***  Overactive Bladder

Another important complication of transobturator tape procedures is the onset of de novo OAB. There is no homogeneity in the data reported in the literature on de novo OAB incidence, ranging from 0% to 38% [18,35]. Moreover, the trend of OAB over time is not clear; Zullo et al. have shown that OAB seems to regress within 12 months. However, other studies with long-term follow-up have reported high percentages of de novo OAB [12,14,15,16,18]. Some studies have also observed an increasing trend in de novo OAB symptoms over time [17]. These findings lead us to consider the multifactorial nature of OAB. It is not only the residual urine caused by bladder outlet obstruction that leads to urgency, but also the irritant stimulus of the tape.

Other factors should also be considered. Firstly, menopausal status appears to increase the rate of OAB in patients undergoing TVT. In addition, prolene tape may induce changes in collagen metabolism leading to paraurethral sclerosis [36]. Therefore, treating voiding dysfunction and reducing urinary retention may also improve OAB symptoms.

Iacovelli et al. found that preoperative abdominal straining during urodynamic evaluation is significantly associated with an increased incidence risk of de novo OAB after surgery [18].

***c.***  Urinary tract infection

Urinary infections after transobturator procedures can be provoked by, on one hand, voiding dysfunction, and on the other, catheterization. These two factors are both related to bladder obstruction. In fact, urinary retention increases the risk of infection, and at the same time, it is treated with catheterization, which can be achieved by clean intermittent self-catheterization (CISC) or an indwelling catheter.

In regard to lower urinary tract infections, Tommaselli et al. noted that UTIs occur in 9.3% of patients [37]. A similar result was observed in the present review, with approximately 10% of patients experiencing UTIs. The percentage of patients with UTIs is higher after surgery but seems to decrease over time. Different findings have been reported for recurrent UTIs, which show moderately elevated percentages even years later. This is likely due to voiding dysfunctions, which could increase the risk of developing UTIs due to urine stasis. For instance, Serati et al. found similar results for voiding dysfunction and recurrent UTIs [14].

***d.***  Tape-related complications

Among the long-term tape-related complications, vaginal erosion or extrusion of the tape are the most significant examples. Vaginal tape erosions can be asymptomatic or symptomatic, causing pain during intercourse or a sensation of a foreign body in the vagina. In the present study, we found a variable incidence of tape-related complication, ranging from 0–7%. However, it is essential to consider that this is a late complication, and most of the studies included in this analysis have a short follow-up period.

The strength of this study lies in being the first systematic review in the literature that focuses on postoperative complications related to transobturator techniques, particularly voiding dysfunctions. Despite that, this study has several limitations. The main limitation is the heterogeneity of the data collected, since all the studies had a broad variability in follow-up time, the methods used to assess objective cure rate, a slightly different definition of voiding symptoms, and a lack of preoperative urodynamic assessment in some studies. In addition, the average follow-up time in several studies was clearly insufficient to assess long-term complications of surgery. Another limitation is that this study was not registered on the International Prospective Register of Systematic Review (PROSPERO). Finally, due to the heterogeneity of study design and outcomes, a formal risk of bias assessment was not possible. Nevertheless, sources of bias were discussed extensively among the authors and are presented.

## 5. Conclusions

This systematic review provides an estimation of the real incidence of voiding dysfunctions and other long-term complications after the transobturator technique.

Voiding dysfunctions are quite common, but they are usually transient and resolve after 12 months. They are often associated with other conditions, such as OAB and DO. The trend of OAB symptoms over time is not clear due to its multifactorial nature. UTIs are also related to voiding dysfunction, as both urinary retention and catheterization can increase the risk of infection. Therefore, treating voiding dysfunctions and reducing urinary retention may improve OAB symptoms and decrease the risk of UTIs.

Finally, since voiding dysfunctions are often transient, adequate preoperative counseling with patients explaining the possibility of developing these complications could enhance the tolerability of these issues and contribute to long-term patient satisfaction. Preoperative counselling should also be accompanied by accurate written informed consent, as written information is easier to assimilate, and re-reading can help with anxiety related to surgery [38].

## Supplementary Materials

The following supporting information can be downloaded at: https://www.mdpi.com/article/10.3390/jcm13164762/s1, Table S1: Results.

## Abbreviations

UI	Urinary incontinence
UTI	Urinary tract infection
LUTS	Low urinary tract symptoms
CISC	Clean intermittent self-catheterization
BOO	Bladder outlet obstruction
SUI	Stress urinary incontinence
UUI	Urge urinary incontinence
MUI	Mixed urinary incontinence
UDS	Urodynamics
OAB	Overactive bladder
DO	Detrusor overactivity
