# Supplementary material for: The True Impact of Voiding Dysfunctions after Transobturator Sub-Urethral Tape Procedures: A Systematic Review of Literature
Source: J Clin Med. 2024 Aug 13;13(16):4762. doi: 10.3390/jcm13164762 (PMC11355201; doi:10.3390/jcm13164762)
Supplement: Supplementary file 1 [file jcm-13-04762-s001.zip › jcm-3119726-supplementary.pdf]

Table S1

Authors	Study type	N°study patients	N° TVT-O/TOT patients	follow-up time	Type of incontinence (TVT-O/TOT)		Success rate (objective) SUI	UPS (pre-op urgency) / OAB/DO	Postoperative complications				Urgency postop improvement/cure (TVT-O/TOT)
					SUI	MUI			Voiding symptoms/ urinary retention	Tape complications (Vaginal erosion, tape exposure/extrusion)	de novo OAB/DO symptoms (including urgency)	Postoperative UTI	
**Abou-Elela et al (2009)**	Prospective cohort study	20	20	6-14 mo (8 mean)	16	n/a	100% negative stress test	n/a	n/a	0	n/a	0	n/a
**Ahn et al (2015)**	Retrospective observational study	467 (449)	449	n/a	449	n/a	n/a	n/a	10 (2.2%) urinary retention 47 (10.5%) voiding difficulty until 3 mo. 63 (14.7%) significant postoperative PVR	n/a	n/a	n/a	n/a
**Angioli et al (2010)**	RCT (TVT vs TVT-O)	72	37	13-69 mo (60 median)	37	0	27 (72.9 %) negative stress test at 60 mo	0	Urinary retention 0% at 60 mo	2 (5.4%) vaginal erosion at 15-16 mo	2 (6.4%) De novo urgency	n/a	n/a
**Aniuliene et al (2009)**	RCT (TVT vs TVT-O)	164	150	12 mo	164	n/a	94.6% negative stress test	n/a	5 (3.3%) Urinary retention at 12 mo	n/a	5 (3.3%) OAB at 12 mo	1 (0.7%) at 12 mo	n/a
**Araco et al (2008)**	RCT (TVT vs TVT-O)	240	120 (20 lost in FU: 100)	12 mo	240	0	83%	0	17 (17%) Re-catheterization	3 (3%) vaginal erosion	3 (3%) detrusor overactivity; 6 (6%) de	n/a	n/a

Table S1

											novo urgency		
**Barry et al (2005)**	prospective observational study (TOT)	83	83	6-8 weeks	83	n/a	81% negative stress test	n/a	4 (4.8%) required catheterization for 2- 4 days	n/a	n/a	n/a	n/a
**Barry et al (2008)**	RCT (TVT vs TOT)	187	79 (80, 1 withdrawn) Monarc TO-SUS (21 lost in FU: 58)	3 mo	79	n/a	84.8%	n/a	5 (8.6%) urinary retention requiring catheter >2 days at 3 mo	3 (5.2%) Sling protrusion at 3 mo	0	9 (15.5%) at 3 mo	n/a
**Charalambous et al (2007)**	Retrospective, comparative study (TVT vs TVT-O)	315	50	4 weeks, 3 mo, 6 mo, 12 mo	50	n/a	94%	n/a	0% urinary retention, 0% dysuria	1 (2%) vaginal erosion, 0% tape extrusion	De novo urgency 8%	4 (8%)	n/a
**Cheung et al (2014)**	Prospective comparative study (TOT vs TVT-O)	213	124 TOT, 89 TVT-O (at 5 years FU: 186)	5 y	213	n/a	82.6% TO-TVT; 82.5% TVT-O	OAB: tot 34.8% -> TOT 39.3%, TVT-O 28.7%. DO: 4.9%-> TOT 5.1%, TVT-O 4.6%	Voiding dysfunctions 2/186 (1.1%): TOT 2/104(1.9%); TVT-O 0% at 5 y	Tape erosion 3/166 (1.8%): TOT 2/92 (2.2%), TVT-O 1/74 (1.4%) at 5 y	De novo OAB 23/186 (12.4%): TOT 11/104 (10.6%), TVT-O 12/82 (14.6%); De novo detrusor overactivity 10/109 (9.2%): TOT 4/69 (5.8%), TVT-O 6/40 (15%); at 5 y	n/a	n/a

Table S1

**Cocci et al (2017)**	Retrospective, multicenter study	219	219	3 mo, 6 mo, 12 mo	219	n/a	83.6% negative stress test	n/a	19 (9.5%) early onset, required catheterization within 24–48 hours after Foley catheter removal	n/a	n/a	n/a	n/a
**H. M. Bianchi-Ferraro et al (2012)**	RCT	122	56 (2 lost in FU:54)	12 mo	56	n/a	87 % at 12 mo	31 (58.5%)	Urinary retention 2 (3.5 %) required catheterization for 5 days	1 (2.7%) tape exposure 12 mo	2 (3.5%)	4 (7.1%) at 12 mo	n/a
**H. M. Bianchi-Ferraro et al (2014)**	RCT (TVT vs TVT-O)	122	56 (2 lost in FU:54)	12 mo, 24 mo	56	n/a	83.9% at 12 mo, 85.7% at 24 mo	31 (58.5%)	Urinary retention 2 (3.5%) at 24 mo	4 (7.1%) vaginal erosion, 3 (5.3%) tape exposure at 24 mo	2 (3.5%) at 24 mo	4 (7.1%) at 24 mo	24 (77.4%) cured at 24 mo
**Huang et al (2012)**	Prospective comparative study (TOT vs TVT-O)	127	67 TOT, 60 TVT-O	3 mo	127	n/a	TOT 95.5%, TVT-O 95% at 3 mo	OAB : TOT 35.8%; TVT-O 33.3%	Worsening /de-novo voiding difficulty: TOT 7 (10.4%), TVT-O 3 (5%) at 3 mo	n/a	OAB symptoms: TOT 9 (13.4%), TVT-O 5 (8.3%)	n/a	n/a
**Huang et al (2018)**	Prospective observational study	78	78	12-15 mo (median 13.5)	78	n/a	76.9% 1-hour pad tes at 6-12 mo	n/a	3 (3.8%) transient urinary retention	1 (1.3%)	De novo OAB 4 (5.1%)	1 (1.3%) transient UTI	n/a
**Huang et al (2020)**	Retrospective cohort study	136	136	12 mo, 5 y	136	0	Negative stress test 126 (92.6%) at 12 mo, 124 at 5 y (91.2%)	0	Voiding dysfunctions: 15 (11.2%) at 12 mo, 14 (10.3% at 5 y)	Sling exposure: 3 (2.2%) at 12 mo, 0 (0.0%) at 5 y	De novo OAB dry 6 (4.4%) at 12 mo, 7 (5.1%) at 5 y.	UTI 4 (2.9%) at 12 mo, 4 (2.9%) at 5 y	n/a

Table S1

**Iacovelli et al (2021)**	Retrospective cohort study	192	192 Group A (abdominal straining at UDS): 60/192 (31.2%); Group B (NO abdominal straining at UDS) 132/192 (68.8%)	3 y	192	0	n/a	n/a	Voiding dysfunctions: group A 9(15%), group B 8(6%) at 3 y	n/a	de novo OAB group A 23 (38%), group B 26 (19.6%)	n/a	n/a
**Karateke et al (2009)**	RCT (TVT vs TVT-O)	164	83 TVT-O	12–16 mo (14 mo mean)	83	n/a	86.7% at 12 months	n/a	Voiding difficulty 6 (7.2%) requiring self-catheterization	Tape erosion 2 (2.4%)	n/a	n/a	n/a
**Liapis et al (2006)**	RCT (TVT vs TVT-O)	91 (2 lost in FU: 89)	43	12 mo	43	n/a	90%	n/a	1 (2.3%) urinary retention	0% vaginal erosion	De novo urgency 13.9% at 12 mo, De novo DO 9.3% at 12 mo	1 (2.3%)	n/a
**Liapis et al (2007)**	RCT (TOT vs TVT-O)	120	65 TVT-O (4 lost in FU:61) ; 55 TOT (2 lost in FU: 53)	12 mo	120	n/a	87% TVT-O, 90% TOT negative stress test and 1h pad test, at 12 mo	n/a	Urinary retention TVT-O 3/61 (5%), 2/53 (3.8%) TOT	0	De novo urgency: TVT-O 13.1%; TOT 11.3%. De novo bladder over activity TVT-O 8.2%, TOT 9.4% at 12 mo	3/61 (5%) TVT-O, 2/53 (3.8%) TOT	n/a

Table S1

**Long et al (2008)**	Retrospective, comparative study (TVT vs TVT-O)	159	68	12 mo	68	n/a	88.2%	n/a	De novo bladder obstruction 1 (1.5%)	4 (5.9%) vaginal erosion	4.4% de novo OAB	10 (14.7%)	n/a
**Melendez-Munoz et al (2018)**	RCT (miniARC vs TVT Abbrevo)	246	125 (5 not allocated intervention, 27 lost in FU: 93). 39 SLING ONLY, 93 ALL	6 mo, 12 mo	82	n/a	ALL: 95.9% (6 mo); 96.0% (12 mo). SLING ONLY 93.5% (6 mo);96.6% (12 mo)	6% DO	At 12 mo: SLING ONLY group: Catheter >1day : 16.7% ALL: Catheter >1day : 35.4%	3 (3.2%) tape exposure at 12 mo	n/a	n/a	n/a
**Meschia et al (2007)**	RCT (TVT vs TVT-O)	231	117 (7 lost in FU: 110)	6 mo median	117	n/a	89% negative stress test at 6 mo	39% OAB	Early post-operative urinary retention 3%, voiding difficulty 4%	n/a	n/a	n/a	n/a
**Montera et al (2018)**	Retrospective observational study	50	50 (7 lost in FU)	1, 5, 10 years	50	n/a	98% at 1 y, 93% at 5 y, 91% at 10 year	n/a	Bladder outlet obstruction 0% at 1,5 and 10 y	2 (5%)	OAB symptoms: 5 (10%) at 1 y, 8 (18%) at 5 y, 10 (23%) at 10 y. DO 0% at 1y, 5y and 10 y. MUI: 0% at 1 y, 1 (2%) at 5 y, 1 (2%) at 10 y	n/a	n/a

Table S1

**Murphy et al (2007)**	Prospective and retrospective cohort study	329 (90 lost in FU--> 239)	232	14.7 mo mean	23 2	n/a	n/a	144 (62.1%) urgency	3 (1.3%) voiding difficulties requiring sling release	0% vaginal erosion	6.6% de novo urgency	n/a	71 (49.2%) urgency resolution
**Neuman et al (2007)**	Retrospectiv e comparative study	150	75	6-13 mo	75	n/a	98.7%	25 (35%) OAB	0% voiding dysfunction	n/a	0% de novo OAB	n/a	n/a

Table S1

**Palva et al (2010)**	RCT (TVT vs TVT-O)	267	131 (5 Lost in FU at 36 mo: 126)	2 mo, 12 mo, 36 mo, 60 mo	131	n/a	89.5% negative stress test at 36 mo	n/a	1 (0.8%) at 12 mo requiring correction)	1 (0.8%) vaginal erosion at 12 mo	7 (5.6%), at 36 mo	16.8% at 12 mo, 17.6% at 36 mo, 5 (around 4%) recurrent infections between 1-3 year FU visit	n/a
**Rechberger et al (2009)**	RCT (TVT vs TOT)	537	268 (71 lost in FU: 197)	1 mo, 4 mo, 6 mo, 12 mo, 18 mo	268	n/a	61.4 %	n/a	Urinary retention 10 (5%), required temporary bladder catheterization	5 (2.5%) tape erosion	De novo OAB: 5%	11 (5.5%)	n/a
**Rinne et al (2008)**	RCT (TVT vs TVT-O)	273	132 (1 lost in FU: 131)	12 mo	132	n/a	93.1%	23 (17.4%)	Retention symptoms 2 (1.5%) requiring catheterization between 2-12 mo	Tape erosion 1 (0.8 %)	De novo urgency 2.3%	22 (16.8%)	n/a
**Ross et al (2009)**	RCT (TVT vs TOT)	199	94 TOT (1 conversion in TVT+ 8 lost in FU: 86, 85 at final appointment)	12 mo	199	n/a	81% pad test at 12 mo	n/a	n\a	68/85 (80%) tape palpable, 5/85 (5.9%) of them with vaginal extrusion of tape, at 12 mo	Urge incontinence 4/86 (4.7%) at 12 months	n/a	n/a

Table S1

**Serati et al (2013)**	prospective cohort study	181	181	1 mo, 3 mo, 12 mo, once per year (26 mo median younger and 25 mo median older patients)	18 1	n/a	91.2% negative stress test at median FU	n/a	Voiding dysfunctions 11 (6.1%) at median FU	1 (0.6%) vaginal erosion at median FU	19 (10.5%) De novo OAB at median FU	11 (6.1%) recurrent UTI at median FU	n/a
**Sola et al (2007)**	Retrospectiv e comparative study	174	98	n/a	98	n/a	100%	n/a	1 (1.2%) early urinary retention (< 7 d) requiring surgery. 1 (1.2%) Late urinary retention requiring surgery	1 (1.02%) tape exposure at 6 weeks	n/a	n/a	n/a
**Teo et al (2011)**	RCT (TVT vs TVT-O)	127	61 TVT-O (8 lost in FU at 6 mo, 32 lost in FU at 1 year)	6 mo, 1 year	12 7	n/a	At 6 mo: 83% (24-h pad test of). At 1 year: 86.2%	n/a	1 (1.6%) requiring intermittent catheterizatio n and then tape division	Tape erosion 1 (2%)	De novo (or worsening) 11.3%	n/a	n/a
**Wang et al (2005)**	RCT (TVT vs TOT)	62 (2 lost in FU TVT group-- > 60)	31 TOT	6-14 mo (9 mo median)	31	n/a	n/a	n/a	Incomplete voiding 4 (12.9%), Dysuria 2 (6.5%), Strain to void 2 (6.5%), Post micturition dribble 1 (3.2%) at 6 mo	n/a	Urgency 3 (9.7%), De novo UUI 1 (3.2%) at 6 mo	n/a	n/a

Table S1

**Wang et al (2009) 1**	RCT (TVT vs TOT)	140	70 TOT	12 mo	70	n/a	92.8% at 12 mo; in isolated SUI 93.75% at 12 mo	n/a	short term voiding difficulties 6 (8.57%), requiring catheterization after surgery	2 (2.9%) tape erosion at 3 mo	De novo Urgency 1.4%, OAB 5.7%	0	n/a
**Wang et al (2009) 2**	RCT (TVT vs TVT-O)	315	155 TVT-O (1 withdrawn, 8 lost in FU: 146)	6 mo, 12 mo, 24 mo, and 36 mo. TVT-O 20.5±10.7 mo median follow-up	155	n/a	6 mo: 91.1% ;12 mo: 89.8 % ; 24 mo: 86.2%; 36 mo: 83.3%	n/a	Urinary retention 4/146 (2.7%)	Tape erosion 3/146 (2.1%)	De novo Urgency 4.1%	n/a	n/a
**Zhang et al (2019)**	prospective cohort study	87	87 (14 lost in FU: 73)	11-13 year (mean 12 y)	87	n/a	82.2%,	0	0% voiding dysfunctions, 16/73 (21.9%) voiding symptoms at 12 y	4 (5.5%) tape exposure at 12 y	9/73 (12.3%) De novo OAB at 12 y	recurrent UTI 6/73 (8.2%) at 12 y	n/a
**Zhu et al (2007)**	RCT (TVT vs TVT-O)	56	27	22-30 mo (27.6 mo median)	27	n/a	92.9%	n/a	No objective evaluation (after catheter removal within 12 h of surgery, many patients complained frequency, dysuria and voiding dysfunction which disappeared within 2 weeks)	0% tape erosion	n/a	n/a	n/a

Table S1

**Zullo et al (2007)**	RCT (TVT vs TVT-O)	72	37	13-21 mo (16 mo median)	37	n/a	89% at 12 mo	0	Postoperative urinary retention 0%	0% tape erosion at 12 mo	OAB symptoms: 3 (8%) at 1 month; 2 (5%) at 6 months; 0% at 12 months.	1 (2.7%) at 12 mo	n/a
**Zullo et al (2019)**	RCT (TVT-O vs TVT-Abbrevo)	158	TVT-O 79, TVT-Abbrevo 79	12 mo, 24 mo, 36 mo	15 8	n/a	TVT-O 87%; TVT-Abbrevo 87%	n/a	Urinary retention (for up to 7 days) TVT-O 1 (1%) TVT-Abbrevo 0 %	tape exposure TVT-O 1 (1%); TVT-Abbrevo 2 (2%)	De novo urgency TVT-O 2 (2%), TVT-Abbrevo 2 (2%)	TVT-O 2 (2%), TVT-Abbrevo 3 (4%)	n/a

LEGEND:
SUI (Stress urinary incontinence)
MUI (Mixed urinary incontinence)
OAB (Overactive bladder)
DO (Detrusor overactivity)
UTI (Urinary Tract infection)
CISC (Clean intermittent self-catheterization)
UUI (Urge urinary incontinence)
UDS (Urodynamics)
RCT (Randomized Controlled Trial)
FU (Follow Up)
Mo (Months)
n/a (Not Applicable)
